# Enhanced Thermostability of Glucose Oxidase through Computer-Aided Molecular Design

**DOI:** 10.3390/ijms19020425

**Published:** 2018-01-31

**Authors:** Xiaoyan Ning, Yanli Zhang, Tiantian Yuan, Qingbin Li, Jian Tian, Weishi Guan, Bo Liu, Wei Zhang, Xinxin Xu, Yuhong Zhang

**Affiliations:** 1 Biotechnology Research Institute, Chinese Academy of Agricultural Sciences, Beijing 100081, China; sjznxy2010@126.com (X.N.); 18770046997@163.com (Ya.Z.); nkyswsnn@163.com (T.Y.); liqingbin2015@sina.com (Q.L.); tianjian@caas.cn (J.T.); GuanWeiShiwork@163.com (W.G.); liubo01@caas.cn (B.L.); zhangwei02@caas.cn (W.Z.); 2 Center for Life Sciences, China Agricultural University, Beijing 100089, China

**Keywords:** glucose oxidase, molecular design, saturation mutagenesis, thermostability

## Abstract

Glucose oxidase (GOD, EC.1.1.3.4) specifically catalyzes the reaction of β-d-glucose to gluconic acid and hydrogen peroxide in the presence of oxygen, which has become widely used in the food industry, gluconic acid production and the feed industry. However, the poor thermostability of the current commercial GOD is a key limiting factor preventing its widespread application. In the present study, amino acids closely related to the thermostability of glucose oxidase from *Penicillium notatum* were predicted with a computer-aided molecular simulation analysis, and mutant libraries were established following a saturation mutagenesis strategy. Two mutants with significantly improved thermostabilities, S100A and D408W, were subsequently obtained. Their protein denaturing temperatures were enhanced by about 4.4 °C and 1.2 °C, respectively, compared with the wild-type enzyme. Treated at 55 °C for 3 h, the residual activities of the mutants were greater than 72%, while that of the wild-type enzyme was only 20%. The half-lives of S100A and D408W were 5.13- and 4.41-fold greater, respectively, than that of the wild-type enzyme at the same temperature. This work provides novel and efficient approaches for enhancing the thermostability of GOD by reducing the protein free unfolding energy or increasing the interaction of amino acids with the coenzyme.

## 1. Introduction

Glucose oxidase (GOD, EC.1.1.3.4) is an aerobic dehydrogenase that catalyses the oxidation of β-d-glucose into gluconic acid and hydrogen peroxide with molecular oxygen as the electron acceptor. In recent years, GOD has become widely used in the food industry, gluconic acid production and in feed additives, in addition to being used as glucose biosensor for medical and environmental monitoring [1,2]. GOD is the most widely-used analytical reagent coupled with catalase for the detection and estimation of glucose in blood and in industrial solutions [3,4]. In baking processes, hydrogen peroxide produced during the catalytic reaction of GOD can oxidise the mercapto group of gluten into disulphide bonds to improve the mesh structure inside the dough, enhancing its elasticity and the quality of the product [5]. GOD can also be exploited to produce calcium gluconate, zinc gluconate, etc. Compared with traditional metal catalysis and fermentation methods for producing gluconate, enzymatic methods have obvious advantages, such as safety, simple operation, and good product quality [6]. GOD is also used as a new feed additive in the feed industry to improve the intestinal acidity of animals to inhibit pathogenic microbial growth. 

Currently commercialized GODs are mainly from *Penicillium* spp. and *Aspergillus niger*. GODs from *Penicillium* spp. possess much higher catalytic efficiency than that of GOD from *A. niger*. The substrate affinity of *Penicillium amagasakiense* GOD is six times higher than that of *A. niger* and the catalytic efficiency (*k*_cat_/*K*_m_) is even more than 10 times higher. However, *Penicillium* GODs exhibited poorer thermostability than that of GOD from *A. niger*. The activity of GOD derived from *P. amagasakiense* decreased rapidly when the temperature exceeded 60 °C [7,8]. The poor thermostability of *Penicillium* GOD has become a key limiting factor preventing its widespread application. Therefore, it is of great practical significance to improve the thermal stability of GOD, especially the GOD from *Penicillium* spp.

The specific structures of proteins are closely related to their thermal stability. Many factors affect the specific structure of proteins, such as amino acid composition characteristics, hydrogen bonds, hydrophobic interactions, electrostatic forces, salt bridges and the stability of loops [9,10,11,12]. Protein structure is the combined effect of these factors in an enzyme molecule, and any changes to these forces may lead to changes in stability. In particular, protein free unfolding energy (ΔG) is one of the most important parameters for the thermodynamic stability of proteins and a main indicator of protein thermostability. The effect of mutations on the thermostability of a protein can be determined by calculating the change in the ΔG value (ΔΔG) after protein mutation, which can be predicted with computer simulations. Molecular dynamics (MD) simulations have been used to identify valuable residues that could lead to significant differences in ΔG. For example, the thermostability of methyl parathion hydrolase from *Ochrobactrum* and lipase B from *Candida antarctica* have been improved substantially using this strategy [13,14], increasing the temperature at which half the protein molecules denature (*T*_m_) of the mutant enzyme by 11.7 °C [13] and 3.6 °C [14] compared to the wild-type, respectively.

In addition to the aforementioned factors, there is a special factor that affects the stability of GOD. GOD is a glycoprotein with a molecular mass of 150–170 kDa from fungi [15,16,17]. It is a homodimer of ∼80 kDa monomers, each of which contains two structural domains, one containing the substrate binding site, and the other housing tightly, but non-covalently, bound flavin adenine dinucleotide (FAD) as a coenzyme. Previous results have suggested that the dissociation of FAD resulted in the destabilized tertiary structures of GOD and it has a critical role in the structure and activity of GOD [18,19]. Therefore, the binding strength of FAD with its surrounding amino acids may also affect the thermostability of GOD.

A *god* gene (GenBank accession number: JN809249) was cloned from *Penicillium notatum* F4 in our previous work, and a codon-optimised *godm* gene has been successfully overexpressed in *Pichia pastoris* [20] and obtained the protein GOD_m_. In the present study, we performed computer-aided design with key amino acid site-directed saturation mutagenesis to improve the thermostability of GOD_m_. The key factors affecting the thermostability of GOD_m_ were analysed at the protein structure level, and two mutants with significantly improved thermostabilities, S100A and D408W, were obtained.

## 2. Results

### 2.1. Selection of Mutant Sites

**Figure 1:**
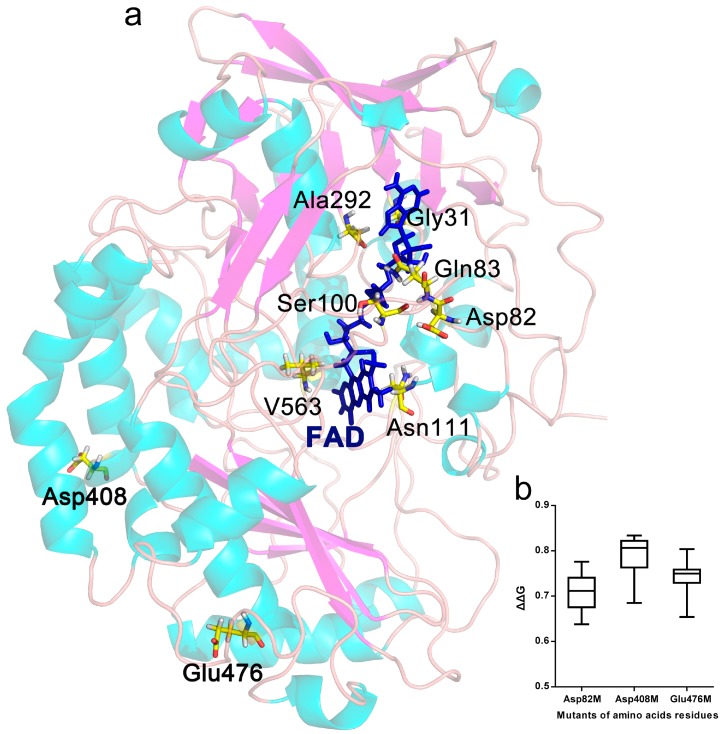
GOD_m_ sites chosen for saturation mutagenesis. (**a**) Ribbon plot of the three-dimensional structure of GOD_m_ from *P. notatum* F4. The selected residues were Asp82, Asp408, Glu476, Gly31, Gln83, Ser100, Asn111, Ala292 and Val563, as shown using the yellow stick model. The blue stick model represented flavin adenine dinucleotide (FAD). The Gly31, Gln83, Ser100, Asn111, Ala292 and Val563 distributed within 5 Å around the coenzyme FAD. The secondary structures of GOD_m_ are colored aqua for helixes and purple for sheets. (**b**). Potential mutant sites whose average of the change in the protein free unfording energy (ΔΔG) values were greater than 0.5.

ΔG is an integrated parameter for the thermodynamic stability of proteins, and the ΔΔG after protein mutation can be predicted with computer simulations. Here, the effects of single point mutations on the ΔΔG of GOD_m_ were evaluated with the Prethermut program [13]. It was considered to exhibit potentially positive effects on protein thermostability when ΔΔG was greater than 0.5 and potentially negative effects when it was below 0.5 [13]. The results showed that the amino acids Asp82 (D82), Asp408 (D408) and Glu476 (E476) could be key sites related to protein thermostability (Figure 1b). The ΔG of most mutants decreased significantly (ΔΔG > 0.5) when these amino acids were mutated into other amino acids, indicative of potentially beneficial mutant positions.

The distribution of all amino acids within 5 Å around the coenzyme FAD in the GOD_m_ structure was analysed with Discovery Studio 2.5 software (Accelrys, San Diego, CA, USA), and the non-covalent interactions between FAD and the surrounding amino acids were analysed. The results showed that mutation of the amino acids Gly31 (G31), Gln83 (Q83), Ser100 (S100), Asn111 (N111), Ala292 (A292) and Val563 (V563) of GOD_m_ potentially enhanced the binding strength between FAD and GOD_m_ due to increased hydrogen and π bonds (Figure 1a and S1).

### 2.2. Mutant Library Construction

Single point mutant libraries corresponding to the nine preselected sites were constructed based on site-directed saturation mutagenesis method. The mutation site was designed with NNK degeneracy (N, adenine/cytosine/guanine/thymine; K, guanine/thymine) to encode all 20 amino acids. The site-directed saturation mutagenesis and homologous recombination in vitro were based on polymerase chain reaction (PCR) and CloneEZ Enzyme. At least 20 colonies from each *E. coli*-mutated library were randomly selected and sequenced with specific primers to detect nucleotide sequences. The mutant rates of the *E. coli*-mutated libraries were above 90%. The resultant recombinant expression vectors were extracted from *E. coli* and transferred into *P*. *pastoris*. After screening based on chromogenic reaction, each mutant library included at least 200 positive transformants.

### 2.3. Thermostable Mutant Selection

The positive transformants were induced, and culture supernatants were collected as crude enzyme solutions since the recombinant GOD_m_s was secretory. The enzyme activity was measured after treatment at 55 °C for 10 min. The transformants with higher residual activities (1.2 times higher than that of the wild-type) were selected as potential candidates. The changed amino acids in the potential candidates were detected through DNA sequencing. Finally, seven mutants (D408W, D408T, D408K, D408C, E476T, E476K and S100A) were obtained from the nine mutant libraries. The crude enzymes of these mutants were purified by anion exchange chromatography. The results of sodium dodecyl sulfate polyacrylamide gel electrophoresis (SDS-PAGE) analysis showed that all of the mutants reached electrophoretic purity (Figure S2). The predicted molecular weights based on SDS-PAGE were around 80 kDa, near the theoretical molecular weight of GOD_m_.

### 2.4. Thermostability and Optimum Temperature of the Mutants

**Figure 2:**
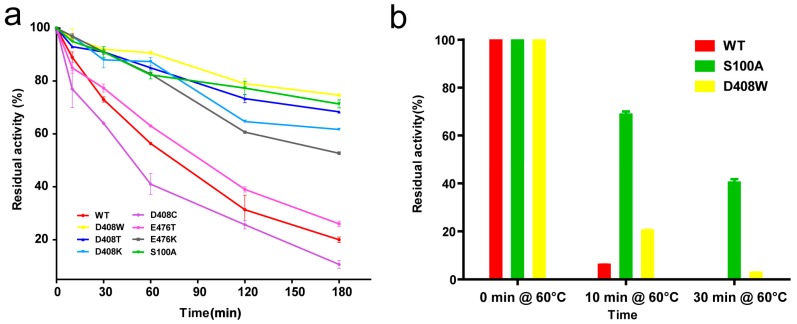

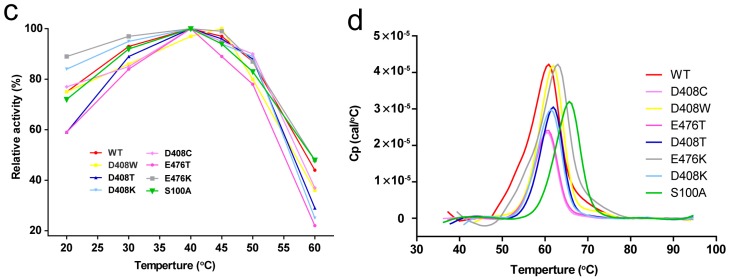
Thermostability of wild-type and mutant GOD_m_. (**a**) Residual activity of the wild-type GOD_m_ and mutants after treatment at 55 °C for different times. Data points correspond to the mean values of three independent experiments. The activity of the untreated enzyme was considered as 100%; (**b**) Residual activities of the wild-type GOD_m_, S100A and D408W after incubation at 60 °C; (**c**) Optimum temperature of the wild-type GOD_m_ and mutants. The highest activities of the mutants and wild-type GOD_m_ were considered as 100%; (**d**) Temperature-induced unfolding measured with differential scanning calorimetry for the wild-type and mutant enzymes. The scans were fitted to a curve after subtraction of an instrument-derived baseline recorded with buffer in both holes as calculated using Origin software (Origin Lab, Northampton, MA, USA).

The protein concentrations of the pure enzyme solutions were standardised to 10 μg/mL, and then treated at 55 °C for 0, 10, 30, 60, 120 and 180 min. The enzyme activity at 55 °C for 0 min was considered as 100%, the specific activities of the wild-type protein, D408W, D408T, D408K, D408C, E476T, E476K and S100A were 55.7, 75.4, 73.2, 66.3, 70.5, 84.3, 52.3 and 85.4 U/mg, respectively. Among the seven mutants, most were more stable than the wild-type protein, especially D408W and S100A. The residual activities of the wild-type, D408W, S100A, D408T, D408K and E476K were 20%, 74%, 72%, 68%, 62% and 53% respectively, after treatment for 180 min (Figure 2a). The half-life (*t*_1/2_)of the wild-type was 75.3 min, while that of D408W was 407.7 min, 4.4-fold higher than that of the wild-type, and that of S100A was 462.1 min, 5.1-fold higher than that of the wild-type (Table 1). The *t*_1/2_ of D408T, D408K and E476K were 315.1, 239.0, 216.6 min, respectively (Table 1). The thermostability of E476T was similar to that of the wild-type. The residual activity of D408C was 10% lower than the wild-type after treatment at 55 °C for 180 min (Figure 2a), and its *t*_1/2_ was 18 min shorter than the wild-type. The thermostability of all the mutants with improved thermostability at 55 °C were further compared with that of the wild-type at 60 °C. After treatment for 10 min, the retention rates of the wild-type, S100A and D408W were 5.6%, 72.8% and 20.8%, respectively. After treatment at 60 °C for 30 min, the wild-type was completely inactivated, while S100A and D408W had 40% and 8.1% residual activities (Figure 2b). However, the other mutants did not exhibit significantly increased thermostability at 60 °C. Furthermore, the optimum temperature of these mutants was 40 °C, showing no difference to the wild-type enzyme (Figure 2c).

### 2.5. Determination of T_m_

*T*_m_ values were determined with differential scanning calorimetry (DSC; GE Healthcare Life Science; Pittsburgh, PA, USA) from 30 °C to 95 °C. The concentration of purified protein was standardised to 200 μg/mL. The measured data were fitted to obtain a smooth bell-type protein dissolution curve (Figure 2d), where the temperature corresponding to the curve peak represented *T*_m_. S100A was the only mutant that showed a significant increase in *T*_m_ among the mutants, as the *T*_m_ value of S100A increased by 4.4 °C compared with the wild-type. The *T*_m_ values of D408K, D408W, D408T and E476K were 0.9–1.3 °C higher than that of the wild-type. Meanwhile, the *T*_m_ of D408C was 0.6 °C lower than that of the wild-type and the *T*_m_ of E476T was similar to the wild-type, with no significant difference (Table 1).

### 2.6. Protein Structure Analysis

**Figure 3:**
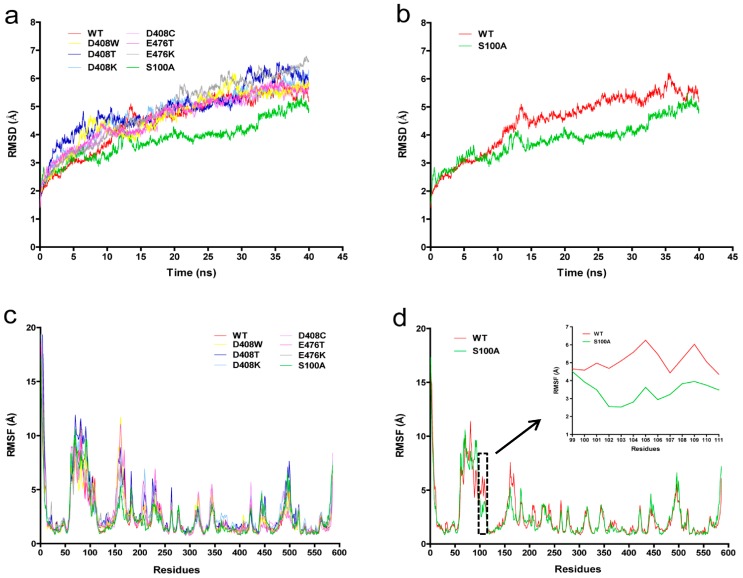
Molecular dynamics (MD) simulation of the protein structure of wild-type and mutant GOD_m_. (**a**) Root mean square deviation (RMSD) values during a 40-ns MD simulation for the wild-type (WT) and mutants at 400 K. The RMSD values of different proteins are shown as different colours; (**b**) RMSD values of S100A and WT; (**c**) Root mean square fluctuation (RMSF) values during a 40-ns MD simulation for wild-type and mutants at 400 K. The RMSF values of different proteins are shown as different colours; (**d**) RMSF values of S100A and WT. RMSF values curve of amino acids located at 99–111 position were boxed in black dotted line and enlarged.

We explored the relationship between protein conformation and thermostability based on an MD simulation. The MD simulations of GOD_m_ were conducted at 400 K to assess the stability of the mutants and the wild-type. Figure 3a shows the root mean square deviation (RMSD) of the backbone atoms of the GOD_m_ mutants. The RMSD tendencies of most mutants were similar to the wild-type. However, those of S100A were steadier than the wild-type at 400 K (Figure 3b). The fluctuation of RMSD values was consistent with the fact that the mutants possessed greater thermostability.

The root mean square fluctuation (RMSF) values reflected the fluctuation of the individual residues during the MD simulation process. The wild-type enzyme had one major unstable region around 51–167 amino acids (Figure 3c). S100 is located in this unstable zone. When serine at position 100 was mutated to alanine, the RMSF value decreased (Figure 3d), a lower RMSF value indicates more stability.

## 3. Discussion

Glucose oxidase is used not only in the food industry and in medical examinations [1], but also as an additive used for monogastric animal feed [2]. However, the poor thermostability of GOD limits its application, especially in the feed industry [21]. Improving the thermostability of GOD can help reduce the loss of activity during the feed pelleting process.

Several approaches have been used to improve the thermostability of GOD in previous studies, including enzyme engineering means such as protein immobilization [22] and protein engineering methods such as directed evolution and rational design. Researchers have improved the thermostability of GOD significantly by directed evolution experiments [23,24]. It is well known that directed evolution is time-consuming and laborious. Alternatively, available protein structure information of GOD makes it possible to obtain thermostable mutants through rational design. Marín-Navarro et al. obtained GOD variants with improved thermostability based on rational design aimed at introducing stabilizing salt bridges [12]. In the present study, amino acids putatively related to the thermostability of the enzyme were predicted through a rational computer-aided molecular design strategy based on ΔΔG and the analysis of amino acids related to the coenzyme FAD, and two mutants with significantly improved thermostability, S100A and D408W, were obtained through site-directed saturation mutagenesis. Our results strongly suggested that rational computer-aided molecular design could be an effective strategy to improve protein thermostability.

We also attempted to investigate the molecular mechanism related to the structural changes and the increased thermal resistance of the mutants. The structural changes in D408W were analyzed with Discovery Studio 2.5, and the results showed that 14 hydrogen bonds could be formed between W408 and other amino acids with distances of 5 Å, which was six more than the number of hydrogen bonds D408 formed in the wild-type protein. These bonds may help maintain the hydrogen bond network around W408 and thereby contribute to the improved thermostability. The RMSD tendency of S100A was steadier and the RMSF values of S100A were markedly lower than those of the wild-type enzyme, demonstrating the enhanced rigidity of S100A. Since the S100A resides in the coenzyme domain, this mutation may contribute to the combination of FAD and GOD_m_, thus improving the stability of the GOD_m_ tertiary structure. Besides, we speculated that when the hydrophilic amino acid S100 was mutated to hydrophobic A100, which is located inside the tertiary protein structure, it helped to increase the stability of the protein.

This work provides novel and efficient approaches for enhancing the stability of GOD_m_ by reducing the ΔG or increasing the interaction between the amino acids with the coenzyme. With markedly improved thermostability, GOD_m_ is better suited for industrial application. In this study, we constructed mutant libraries based on only one mutant site to test the effectiveness of the computer-aided molecular design strategy. To obtain even greater thermostability of this enzyme, we will attempt to construct and screen GOD_m_ mutants with combinations of two mutation points in future research.

## 4. Materials and Methods

### 4.1. Strains, Plasmids, and Media

*Escherichia coli* Trans1-T1 (TransGen, Beijing, China) was used for the gene cloning. *Pichia pastoris* GS115 (Invitrogen, Carlsbad, CA, USA) was used as the host for recombinant GOD_m_ expression. The *P. pastoris-E. coli* shuttle expression vector pPIC9-*godm* was previously constructed by our laboratory [20]. Luria-Bertani (LB) medium, regeneration dextrose base (RDB) medium, minimal methanol (MM) medium, and yeast peptone dextrose (YPD) medium were prepared according to the instructions of the *Pichia* expression kit (Invitrogen, Carlsbad, CA, USA). Fermentation basal salts (FBS) and PTM trace salts were prepared in compliance with the *Pichia* fermentation process guidelines (Invitrogen, Carlsbad, CA, USA).

### 4.2. Mutant Site Selection

The protein structure model of GOD_m_ was constructed with SWISS-MODEL (https://www.swissmodel.expasy.org/) using *Penicillium amagasakiense* GOD (PDB accession 1GPE) [25] as a template. An MD simulation was performed on the 1GPE model as the starting structure to select residues with high RMSF values for mutation to improve protein thermostability [26]. Dynamics and trajectory analyses were performed with the software NAnoscale Molecular Dynamics (NAMD) using the charmm22 force field. An appropriate amount of NaCl was added to neutralise the system. The LINCS algorithm was used to constrain bond lengths and a 2-fs time step was used. For the simulations at 400 K, production runs were performed for 20 ns with a 2-fs step. Temperature and pressure coupling were obtained with v-rescale [27,28]. 

The ΔΔG of GOD_m_ was calculated using the software Prethermut. All possible single-point saturation mutants of GOD_m_ with ΔΔG values above 0.5 compared with the wild-type were selected [29]. The amino acid residues were selected based on the RMSF and ΔΔG values. Discovery studio 2.5 software was used to determine the distribution of all amino acids within 5 Å of the coenzyme FAD in the GOD_m_ structure. The surrounding amino acids that could affect the binding intensity were analysed.

### 4.3. Plasmid Construction

The mutant plasmids were constructed based on a DNA assembly cloning kit (CloneEZ^®^ PCR Cloning Kit, Genscript, Nanjing, China), which could assemble DNA fragments with varied overlaps (15–80 bp) (construction steps are shown in Figure S3). The strategy involved two pairs of partially overlapping primers (Bgl II-F and Bgl II-R, special F and special R), with 20–25-nt overlapping regions at the 5′-ends of the primers. Primer was designed with a mutant base NNK (N, adenine/cytosine/guanine/thymine; K, guanine/thymine) which was located in the middle of the special F [13] (Table S1). Fragment 1 (using Bgl II-F and special R primer) and fragment 2 (using special F and Bgl II-R primer) were amplified from the pPIC9-GOD_m_ plasmid with FastPfu DNA Polymerase (TransGen, Beijing, China) with PCR. The PCR parameters were as follows: denaturation at 94 °C for 5 min, 30 cycles of 30 s at 94 °C, 30 s at 58 °C, and 1 min at 72 °C, followed by 10 min at 72 °C. Two DNA fragments were homologous to each other, one of which had a mutant base. The fragments were purified using a PCR purification kit and then combined using CloneEZ^®^ PCR Cloning Kit.

The recombinant plasmids were transformed into *E. coli* Trans1-T1. The colonies grew on LB solid medium containing ampicillin, and single colonies were randomly selected from the LB solid medium and DNA from the colonies was sequenced with specific primers to confirm the nucleotide sequences of the mutated sites. The sequencing results were analysed to ensure that the mutant rate was higher than 90%. All transformants were scraped and inoculated into LB liquid medium. Mixed plasmids were extracted after culturing at 37 °C for 12 h.

### 4.4. Positive Transformant Screening

The mixed plasmids were linearised with *Bgl*II, and then transformed into *P. pastoris* GS115 competent cells by electroporation. Transformants were cultivated on RDB plates at 28 °C for 48 h, and then inoculated and incubated on MM plates for another 24 h. The positive transformants were screened by chromogenic reaction for 30 min at room temperature [30]. Individual positive transformants were placed in 48-well microtiter plates containing 500 μL of buffered glycerol complex (BMGY) medium, and then incubated at 28 °C for 48 h with 200 r/min of shaking. The transformants were cultivated further in buffered methanol complex medium (BMMY) at 28 °C for 48 h after the BMGY medium was discarded by centrifugation. The enzyme solutions were harvested by centrifugation and then transferred into 96-well plates. The enzyme solutions were heated to 55 °C for 10 min and then immediately placed on ice. The treated samples were used for the enzyme activity assay. Transformants with high residual activity were selected as potentially positive mutants. The mutant gene sequences of the transformants were amplified by PCR and sequenced.

### 4.5. Protein Expression and Purification

The cells were cultured in 200 mL of YPD medium in a 500-mL shake flask for 48 h. The cells were harvested by centrifugation, and then resuspended in 200 mL of FBS (where glucose acted as the sole carbon source) for 24 h. Finally, the cells were harvested by centrifugation and resuspended in 100 mL of FBS with 1% methanol for 72 h. The culture supernatant was harvested by centrifugation at 8000 g/min for 10 min. The above crude enzyme solution was desalted and purified according to the methods described previously [31]. The protein purity was evaluated with SDS-PAGE.

### 4.6. Enzyme Assay

Glucose oxidase activity was measured following the method described previously [20].

### 4.7. Thermostability Assay

Enzyme thermostability was characterised with *o*-dianisidine, d-glucose, and horseradish peroxidase as the substrates. The enzyme solution was treated at 55 °C for 0, 10, 30, 60, 120 and 180 min and the activity was measured under standard conditions. The activity of the untreated enzyme was considered as 100%, and mutants with high residual activities were selected for subsequent analysis. The optimal temperature for GOD_m_ activity was determined based on the standard enzyme assays in the range of 20–60 °C in 100 mM NaH_2_PO_4_/Na_2_HPO_4_ buffer (pH 6.2).

The half-lives of the wild-type and mutant enzymes were determined by incubation in water baths at 55 °C. Samples were incubated for various times, and then placed immediately on ice. The residual activities of the samples were measured using a standard assay, where *t*_1/2_ = ln2/K_D_. The rate constant, K_D_, was calculated using GraphPad Prism software (GraphPad Software, Inc., La Jolla, CA, USA).

The *T*_m_ was measured to indicate the stability of protein unfolding. The protein concentration was standardised to 200 μg/mL. DSC was used to measure *T*_m_. The samples were diluted in 0.02 M sodium phosphate buffer (pH 6.0) and heated from 30 °C to 95 °C. Each sample was tested three times in 96-well plates.

### 4.8. Mutant Protein Structure Analysis

The mutant GOD_m_ was evaluated with an MD simulation with PyMOL (Delano Scientific, San Carlos, CA, USA) and Discovery Studio 2.5 software. The RMSD and RMSF values were measured. The key amino acids affecting the thermostability of GOD_m_ were revealed through the protein structures analysis.

## Figures and Tables

**Table 1:** Thermostability of the wild-type and mutant enzymes.

Mutant	*t*_1/2_ (min) at 55 °C	*T*_m_ (°C)
WT	75.3	60.7
D408W	407.7	61.9
D408T	315.1	61.7
D408K	239.0	61.6
D408C	57.3	60.1
E476T	110.0	60.9
E476K	216.6	62.0
S100A	462.1	65.1

*t*_1/2_ = ln2/K_D_, where the rate constant K_D_ is the time to reach the abscissa in the plot, ln (U_t_/U_0_) is the ordinate, U_t_ is the time t of enzyme activity, and U_0_ is the time 0 of enzyme activity.

## Supplementary Materials

Supplementary materials can be found at http://www.mdpi.com/1422-0067/19/2/425/s1.
