# Supplementary material for: Enhanced Thermostability of Glucose Oxidase through Computer-Aided Molecular Design
Source: Int J Mol Sci. 2018 Jan 31;19(2):425. doi: 10.3390/ijms19020425 (PMC5855647; doi:10.3390/ijms19020425)
Supplement: Supplementary file 1 [file ijms-19-00425-s001.pdf]

Table S1. Sequences of the primers used for site-directed mutagenesis.

Mutant library name		Primer sequence (5'–3')
	Bgl II-F	CAAGATCTCTGCCTCGCGCGTTTC
	Bgl II-R	GAAACGCGCGAGGCAGAGATCTTG
D82	D82F	GAAATCTTCGGAACTTCTGTTNNKCAAAAC
	D82R	AACAGAAGTTCCGAAGATTTCACC
D408	D408F	CTACAGAAACTGGTTGCTGNNKGAGG
	D408R	CAGCAACCAGTTTCTGTAGTTCTCG
E476	E476F	GCCACTAAGTTGGGTAGANNKTTG
	E476R	TCTACCCAACTTAGTGGCAGC
G31	G31F	TAAAACTTACGACTACGTCATTGCTNNKGGAGGCCT
	G31R	AGCAATGACGTAGTCGTAAGTTTTACCCT
Q83	Q83F	TGAAATCTTCGGAACTTCTGTTGACNNKAACTACTT
	Q83R	GTCAACAGAAGTTCCGAAGATTTCACCGT
	S100F	ACAATAGAACTGGTGAGATTAAGNNKGGATTGGG
S100	S100R	CTTAATCTCACCAGTTCTATTGTTGAT
N111	N111F	GGCCTTGGTGGTTCCACCTTGATCNNKGGTGACAG
	N111R	GATCAAGGTGGAACCACCAAGGCCCAATCC
A292	A292F	ACGCCAAGCAAGAAGTTTTGCTGNNKGCCGGTTC
	A292R	CAGCAAAACTTCTTGCTTGGCGTAGACATTG
V563	V563F	TCCCTCCAACTCAAGTTTCCTCTCACNNKATGACCGT
	V563R	GTGAGAGGAAACTTGAGTTGGAGGGATAGAA

N represents A, G, T, or C. K represents G or T.

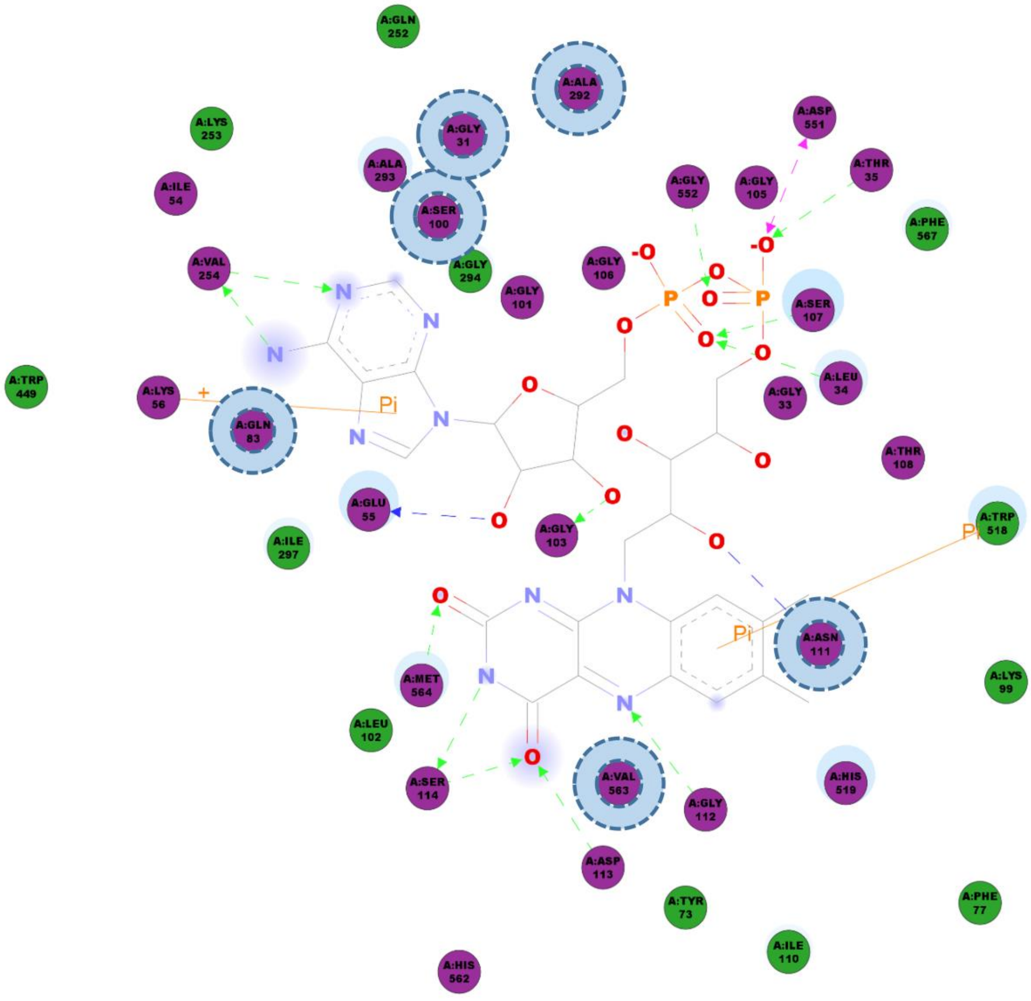

Figure S1. Interaction between FAD and surrounding amino acids in the GODm protein. Gly31, Gln83, Ser100, Asn111, Ala292, and Val563 were predicted with Discovery Studio 2.5 software (as shown with blue dotted lines).

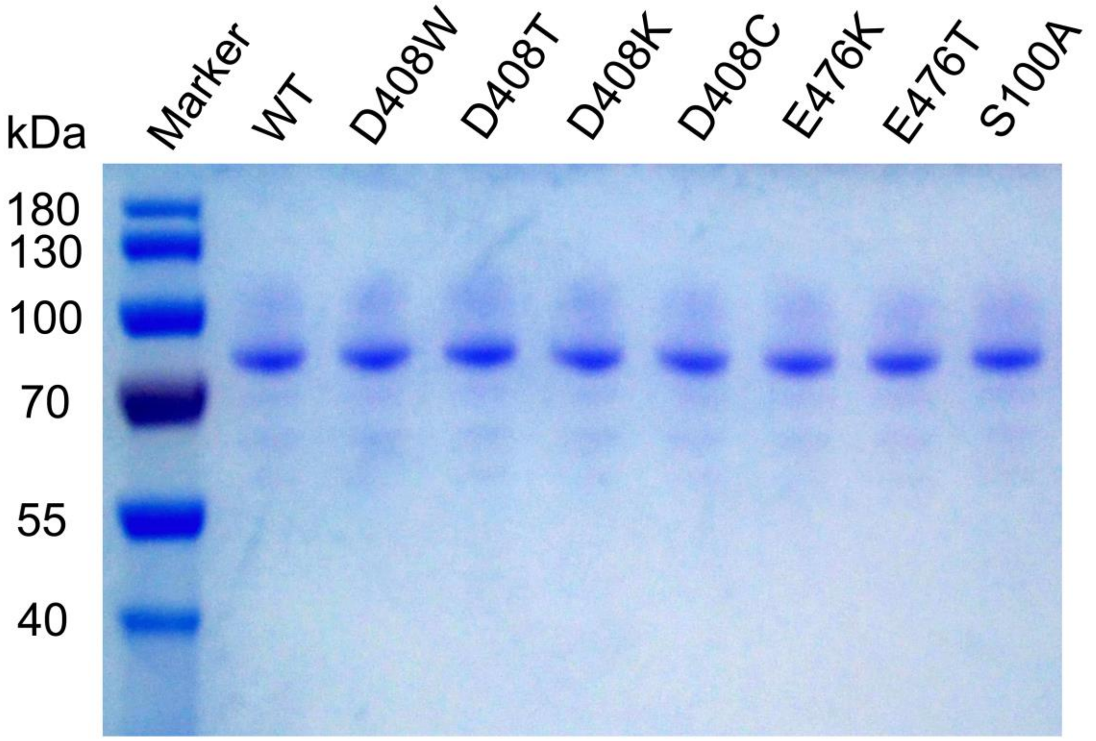

Figure S2. SDS-PAGE analysis of purified wild-type and mutant GOD$_m$. M, marker; WT, purified wild-type GOD$_m$; D408W, D408T, D408K, D408C, E476T, E476K, and S100A were the purified mutant proteins.

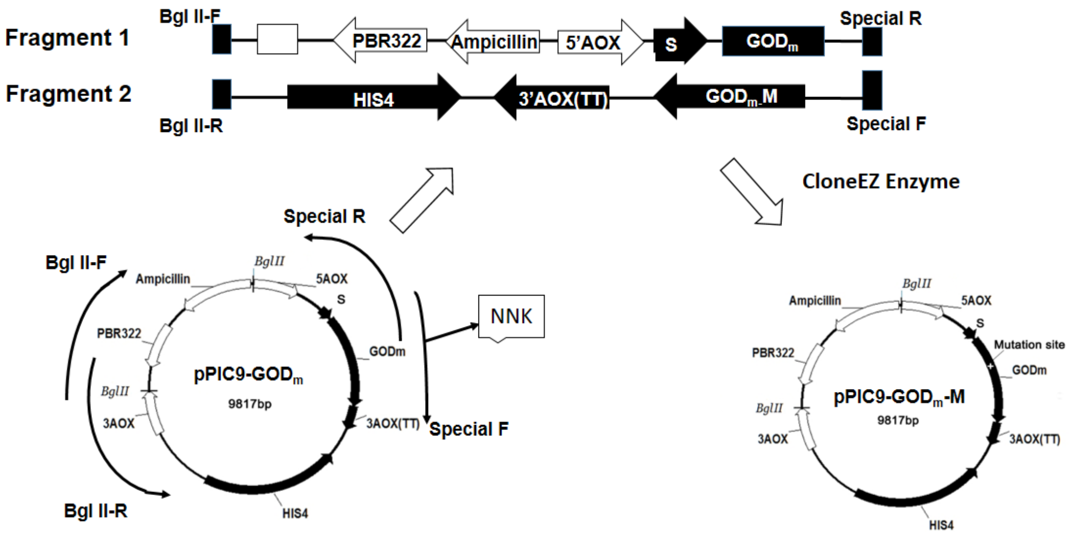

Figure S3. The schematic diagram of mutant plasmids construction. pPIC9-GODm was used as the template plasmid and pPIC9-GODm-M represented the mutant plasmids.
